# Canada Veterinary Associations

**Published:** 1898-07

**Authors:** 


### AMONG THE PROFESSION IN CANADA.

### CANADA VETERINARY ASSOCIATIONS.

*Ontario Veterinary Medical Association.*—Secretary, C. H. Sweetapple, 46 Temperance Street, Toronto, Ontario. Meetings, March and September.

*Manitoba Veterinary Association.*—Secretary, W. A. Dunbar, Winnipeg, Manitoba. Meetings annually, February.

*Western Ontario Veterinary Medical Association.*—President, William Gibbs, St. Mary's, Ontario.

*Ontario Veterinary Medical College Society.*—Secretary, C. W. Fisher, Cabot, Vt. Meetings bi-weekly during college term.

*Montreal Veterinary Medical Association.*—Secretary, W. B. Wallis, 6 Union Avenue, Montreal. Meetings monthly during college term of McGill Veterinary Department.

